# The impact of non-stereotypical gender role endorsement in live broadcasting on consumers’ purchase intention

**DOI:** 10.3389/fpsyg.2024.1359952

**Published:** 2024-03-19

**Authors:** Jia Fu, Simin Huang, Xiaolin Chen

**Affiliations:** School of Journalism and Communication, South China University of Technology, Guangzhou, China

**Keywords:** non-stereotypical endorsement, gender role, gender ideology, scheme theory, e-commerce live broadcasting

## Abstract

**Introduction:**

Non-stereotypical gender role endorsement is becoming more common in e-commerce live broadcasting. However, there is relatively little research on this topic, and the mechanism of its impact on purchase intention is not yet clear. Based on schema theory and experimental methods, this study explores the impact of non-stereotypical gender role endorsement (compared to stereotypical gender role endorsement) on purchase intention in e-commerce live broadcasting. Besides, we take traditional gender ideology as the moderating variable.

**Methods:**

We first selected experimental materials available for formal experiments through two pre-experiments. Secondly, this study conducted experiments on male/female product groups, respectively. Participants were recruited through the Credamo platform for both experiments.

**Results:**

Experiment 1 indicates that for female product, stereotypical gender role endorsement triggers higher consumer purchase intention compared to non-stereotypical gender role endorsement. The subsequent moderating effect test results manifest that traditional gender ideology plays a moderating role in this effect. Experiment 2 shows that for male product, there is no significant difference in the impact of the two types of endorsement on consumers’ purchase intention. In other words, non-stereotypical gender role endorsement does affect consumers’ purchase intention, but this effect exists only in female product, and is more significant for consumers with a high level of traditional gender ideology.

**Discussion:**

This study not only has certain theoretical significance for expanding the application boundaries of schema theory and congruence between celebrities and products endorsed, but also has practical significance for brand owners and streamers to effectively adopt non-stereotypical gender role endorsement to enhance purchase intention.

## 1 Introduction

Celebrity endorsement is one of the most popular marketing methods today (Halonen-Knight and Hurmerinta, 2010). With the rapid development of online technology comes the rise of live-stream sales. Live e-commerce platforms have opened up new channels for celebrity endorsements (Park and Lin, 2020). It was showed that the characteristics of streamers could influence consumers’ purchase intention (Guo et al., 2022; He et al., 2022; Chen and Yang, 2023; Yang et al., 2023). As one of the characteristics, gender plays an important role and will largely influence the marketing effectiveness (Hudders and De Jans, 2022).Thus, the gender of the streamer is one of the important factors to be considered in endorsement marketing.

In the field of e-commerce, it has been shown that the congruity of the sales assistant’s gender and the product’s gender positively affects the consumer’s purchase intention (Beldad et al., 2016). However, there is an interesting trend in celebrity endorsement campaigns in recent years, where male spokespersons endorse products with female gender images or female spokespersons endorse products with male gender images. For instance, Austin Li, a male Chinese streamer, has become the most popular spokesman for skincare and cosmetic products in the live e-commerce platform operated by Taobao.com (Yang et al., 2023). This phenomenon is also known as Non-stereotypical Gender Role Endorsement.

Nowadays, it is very common for male (female) streamers to sell female (male) oriented products (Wang and Dong, 2022). Current research focuses on the impact of an streamer’s gender role (single gender/androgyny) on consumer product preference (Wenting et al., 2022). Some studies have also examined the effectiveness of male streamers in cross-gender endorsement and then confirmed that female consumers feel gender identity incongruence due to male streamers’ endorsement of female products, which in turn negatively affects their purchase intention (Chen et al., 2024). Nonetheless, for e-commerce live broadcasting of male products, existing studies have not thoroughly explored whether non-stereotypical gender role endorsement affects consumers’ purchase intention. In addition, even the studies on female products have not clearly figured out the impact mechanism in depth by combining consumers characteristics such as gender role concepts.

In fact, the matching relationship between endorser gender and product gender has been fully explored by relevant studies on traditional marketing channels. However, e-commerce live broadcasting, a novel cyber-commerce amalgamation of live video and online shopping (Liu, 2020), has brought about disruptive changes in terms of sales logic, product presentation, social attributes, etc. (Qin et al., 2023). Differences between e-commerce live broadcasting and traditional marketing channels underscore the need for dedicated research in this domain (Wongkitrungrueng and Assarut, 2020). Furthermore, even in studies on traditional marketing channels, the effectiveness of non-stereotypical gender role endorsement has not yet reached a consensus conclusion. It may have a positive or negative impact on purchase intention (Åkestam et al., 2021; Hudders and De Jans, 2022; Wang and Dong, 2022). This inconsistency in findings calls for research that can test whether the impact of non-stereotypical gender role endorsement is positive or negative.

Psychological research has shown that there is a match or mismatch between the gender of the endorser and the gender image of the product, which can be explained by schema theory (Lynch and Schuler, 1994). Besides, individuals’ gender ideology influences their perceptions of stereotypical gender role endorsement and non-stereotypical gender role endorsement (Cheung and Choi, 2016; Apgar and McManus, 2019). Thus, in order to fill the research gap mentioned above, the current study aims to explore the impact of non-stereotypical gender role endorsements (compared with stereotypical gender role endorsements) in e-commerce live broadcasting, especially the influence on purchase intentions for products with male and female gender images. Additionally, we seek to assess how traditional individual gender role concepts moderate this effects.

## 2 Theoretical framework and hypotheses development

### 2.1 Gender ideology and gender stereotypes

Gender role is the profile of an individual’s gender-typed characteristics, attitudes, and interests (Garnets and Pleck, 1979). It has also been argued that gender roles refer to the normative expectations that exist for gender division in social interactions within a particular historical or cultural context (Gilbert, 1985). In addition, gender ideology encompasses societal beliefs about gender roles, norms, and relationship patterns for men and women. Following the Dualism of gender roles, gender roles can be categorized into a single male or female dimension, thus creating distinct gender stereotypes (Grau and Zotos, 2016).

Gender stereotypes are defined as beliefs that distinguish different genders based on certain characteristics, attributes, and behaviors (Eisend, 2010), which prescribe what men and women should be like and how they should behave in different areas of their lives (Ellemers, 2018). Gender stereotypes have been shown to have an impact on people’s judgments and attitudes in a variety of contexts. In the context of marketing, gender stereotypes have been shown to influence consumers’ attitudes toward advertisements, attitudes toward products, purchase intentions, and other aspects (Eisend et al., 2014; Åkestam et al., 2021; Gordon and Furnham, 2021; Lucka et al., 2021). In addition, products can be made gender-specific by combining gender stereotypes with product attributes (Stern et al., 1993).

### 2.2 Non-stereotypical gender role endorsement and scheme theory

Prior research has shown that gender stereotypes can be used in advertising in various ways (Furnham and Paltzer, 2010), stereotypical gender role endorsement is one of the common ways. Stereotypical gender role endorsement emphasizes matching the gender of the spokesperson with the gender attributes of the product (Lien et al., 2012). For example, men are supposed to endorse products with male gender images such as cars, beer, etc. Conversely, non-stereotypical gender role endorsement means that the spokesperson does not conform to the gender stereotypes or even shows the opposite expectation or behavior in the endorsement activities (Luoh and Lo, 2012; Lo et al., 2020), such as women endorsing cars and other products with male images in traditional concepts. In other words, one of the two endorsement patterns reflects the congruence between the gender of the spokesperson and the perceived product gender, while the other reflects the incongruence, which can be explained by schema theory (Lynch and Schuler, 1994).

According to schema theory, schema refers to a cognitive or knowledge structure that previously existed in memory (Wright, 1986). The gender image of a product, as prior knowledge stored in a consumer’s schema, influences how consumers process and understand current marketing-related information (Lien et al., 2012). In fact, stereotypical gender role endorsement and non-stereotypical gender role endorsement, respectively, reflect the match and mismatch between the gender of the endorser (novelty) and the gender image of the product (schema) (Lynch and Schuler, 1994), which in turn affects consumers’ responses to brands, endorsers and advertisements.

### 2.3 Hypothesis development

Both stereotypical gender role endorsement and non-stereotypical gender role endorsement can affect consumers’ purchase intentions (Åkestam et al., 2021; Chen et al., 2024). When the endorser’s gender matches with the product’s gender image, schema congruence is generated (Meyers-Levy and Tybout, 1989). Conversely, schema incongruence was produced when the endorser’s gender did not match the product’s gender image. With regard to which situation leads to better results, most studies have concluded that schema congruence has a more positive impact (Meyers-Levy and Tybout, 1989; Wang and Dong, 2022). Even though some studies have argued that schema incongruence, especially moderate incongruence, produces better results, the production of such effects requires controlling for boundary conditions such as brand awareness, consumer product involvement, etc. (Mandler, 1982; Debevec and Iyer, 2013; Åkestam et al., 2021). Moreover, a recent research on cross-gender endorsement of live e-commerce broadcasting also suggests that endorsement of female products by male streamers (non-stereotypical gender role endorsement) negatively affects purchase intentions (Chen et al., 2024).

Notably, the impact of male endorsing female-oriented products and vice versa is asymmetric (Whipple and McManamon, 2002). Studies indicate that the effects of stereotypical and non-stereotypical gender role endorsement on products with a female gender image is different. Nevertheless, the difference is negligible for products with male gender images (Whipple and McManamon, 2002). One aim of our study is to further explore the above asymmetrical effects. In other words, is there a significant difference in the impact of the two endorsement modes on purchase intentions? There is no definitive conclusion. Therefore, Hypothesis 1 and 2 can be proposed as follows:

*H1*: Stereotypical gender role endorsement triggers higher purchase intentions than non-stereotypical gender role endorsement for products with female gender images.

*H2*: There is no significant difference between the effect of stereotypical gender role endorsement and non-stereotypical gender role endorsement on consumers’ purchase intentions for products with male gender images.

Besides, higher gender ideology leans toward traditional views, emphasizing distinct roles played by men and women, while lower gender ideology supports a more balanced and equal perspective (Greenstein, 2000; Cheung and Choi, 2016; Apgar and McManus, 2019). There is also evidence that individuals’ gender ideology influences their perceptions of stereotypical and non-stereotypical gender role endorsements. Those with high gender ideology tend to favor stereotypical gender role endorsements over non-stereotypical gender role endorsements, while those with lower gender ideology may not exhibit significant differences in their attitudes toward both types of endorsement (Morrison and Shaffer, 2003; De Meulenaer et al., 2018). As women’s societal status rises, most individuals’ attitudes toward gender roles tend to be more open, but they may not necessarily abandon gender stereotypes (Hudders and Vyncke, 2013). In contrast, in e-commerce live broadcasting research, gender ideology moderates the effect of non-stereotypical gender role endorsements by streamers on consumers’ product preferences (Wenting et al., 2022). Thus, Hypothesis 3 and its sub-hypotheses of this study can be formulated as follows:

*H3*: Consumers’ traditional gender ideology moderates the effect of the endorsement type (stereotypical/non-stereotypical gender role endorsement) on purchase intentions.

*H3a*: For consumers with high traditional gender ideology, their purchase intentions for products with stereotypical gender role endorsement will be higher compared to non-stereotypical gender role endorsement.

*H3b*: For consumers with low traditional gender ideology, there is no significant difference in purchase intentions for products with stereotypical gender role endorsement or non-stereotypical gender role endorsement.

## 3 Methodology

### 3.1 Research design

This study included two pre-experiments and two formal experiments. The pre-experiments aimed to identify products with typical female/male gender images (Pre-experiment 1) and to recognize representative female/male streamers (Pre-experiment 2). Based on the results of pre-experiments, formal experiments tested the effect of using non-stereotypical gender role endorsement on consumers’ purchase intentions for products with female (Study 1) and male (Study 2) gender stereotypes in live broadcasting, and further examined the moderating effect of traditional gender ideology. The data in this section are available in the (Supplementary materials).

### 3.2 Pre-experiment 1

In order to screen out the testing materials in subsequent experiments, Pre-experiment 1 was designed to identify products with typical female/male gender images.

#### 3.2.1 Material and procedure

Firstly, male and female products to be tested were selected. With comprehensive reference to findings of existing studies (Milner and Fodness, 1996; Fugate and Phillips, 2010) and the GMV data of e-commerce live broadcasting on Taobao, a popular online shopping and retail platform in China, four products with female gender images, namely shampoo, lipstick, body wash and cleanser, and three products with male gender images, namely beer, coffee and razor, were initially selected as the test materials.

Subsequently, 72 students were recruited from a university in southern China to complete Pre-experiment 1 (*N* = 72, female 51.39%, age under 25). These participants were randomly assigned into either the male or female product group. In order to ensure that participants could recall their memories of the products, we asked them to view pictures of the products and then to rate how masculine or feminine the products were. 37 students were randomly assigned to the female product group and viewed pictures of shampoo, lipstick, body wash, and face cleanser, while 35 students were randomly assigned to the male product group and viewed pictures of beer, coffee, and razor. After viewing the pictures, participants rated the masculinity/femininity of each of the products they saw. Due to copyright issues, we did not present the pictures used in our experiment design. Similar images can be obtained by searching the product name on material websites such as vcg.com.

Finally, the gender identity scores for each product were calculated and then the representative male/female products were selected.

#### 3.2.2 Measurement

Participants were asked to rate how masculine or feminine the product felt (1 = none of this trait, 7 = very much of this trait). For products with female (male) gender characteristics, its male (female) gender characteristics score is subtracted from its female (male) gender characteristics score to obtain its gender characteristics score (Chu et al., 2016).

#### 3.2.3 Analysis and results

Paired-samples *t*-tests were then conducted to test whether there were significant differences in the gender characteristics of the 7 products. The testing results showed that the feminine characteristics of lipstick [*M*_femininity_ = 4.95, *M*_Masculinity_ = 2.78, *t*(1,36) = 7.422, *p <* 0.01] and facial cleanser [*M*_femininity_ = 4.70, *M*_Masculinity_ = 3.14, *t*(1,36) = 6.433, *p* < 0.01] were significantly higher than the masculine characteristics. Lipstick had a gender trait score of 2.17, while facial cleanser had a gender trait score of 1.56. In addition, beer [*M*_femininity_ = 3.40, *M*_Masculinity_ = 5.63, *t*(1,34) = 5.759, *p* < 0.01] and razors [*M*_femininity_ = 1.49, *M*_Masculinity_ = 6.77, *t*(1,34) = 30.758, *p <* 0.01] are both significantly more masculine than feminine. Beer had a gender trait score of 2.23 and razors had a gender trait score of 5.28. Since the frequency of razor and facial cleanser of the same brands and models endorsed by different male and female streamers is less and does not have a high degree of generation, razor and facial cleanser are not used as experimental products in this study. Finally, this study selected lipstick as a product with a female gender image, and beer as a product with a male gender image.

### 3.3 Pre-experiment 2

Pre-experiment 2 was designed to select streamers with typical female and male traits, which in turn led to the screening of streamer video experiment materials for the formal experiment phase.

#### 3.3.1 Material and procedure

First of all, the streamers that can be used as experimental materials were selected. Referring to the streaming rankings on the “Firefly” Taobao live third-party data platform, the top 50 streamers were selected according to the dimension of “highest PV per hour.” Combined with the richness of each streamer’s recorded material, 4 male streamers, and 3 female streamers, were selected for experiments. Afterwards, the live broadcast scenes of the 7 streamers were edited into about 1-min videos, respectively (The videos is from https://taolive.taobao.com). Among them, the streamers in the female product group endorsed lipstick products, and the streamers in the male product group endorsed beer products.

Subsequently, the recruitment of experimental participants for the female product group and the male product group was, respectively, conducted on the Credamo platform. For the female product group, a total of 52 participants were recruited (*N* = 52, female 59.62%, age from 18 to 40), and subjects were randomly assigned to watch videos of lipstick endorsed by either male streamers or female streamers. All participants were then asked to rate the gender and other characteristics of the streamers in each video.

For the male product group, 50 participants were recruited (*N* = 50, female 50.00%, age from 18 to 60). The subjects were randomly assigned to watch a video of the male streamer, or female streamer, endorsing a beer product. And then all participants were asked to rate the gender traits and other characteristics of the streamers in the video.

To ensure that all recruited participants have carefully watched the video, we set a screening question, what was not mentioned in the video. Based on the answer results, participants who did not watch the video carefully and answered incorrectly were eliminated. Finally, the feature scores of the streamers were calculated to, respectively, select the representative male/female streamers in male/female product group.

#### 3.3.2 Measurement

In order to evaluate the masculinity and femininity of the streamer, participants were asked to choose their answers to the questions “The streamer has typical masculine traits” and “The streamer has typical feminine traits” from a list of 7 levels of agreement (1 = not at all agree, 7 = strongly agree) (Chu et al., 2016). At the same time, participants were asked to answer questions about five characteristics of the streamer: familiarity, likability, professionalism, trustworthiness, and attractiveness in order to exclude the influence of factors other than gender traits on the results (Ohanian, 1991). Each of these five characteristics is measured using the 7-point Likert Scale.

#### 3.3.3 Analysis and results

We used independent sample t-tests for difference testing. The t-test results showed that in the female gender image products group, the female streamer 1 had a significantly higher score of femininity than the male streamer 1 (*M*_Female streamer 1_ = 5.67, SD_Female streamer 1_ = 1.04; *M*_Male streamer 1_ = 3.12, SD_Male streamer 1_ = 1.54; *p* < 0.01). Male streamer 1’s masculinity score was significantly higher than that of the female streamer 1 (*M*_Male streamer 1_ = 4.36, SD_Male streamer 1_ = 1.38; *M*_Female streamer 1_ = 2.59, SD_Female streamer 1_ = 1.34; *p* < 0.01). Therefore, in the female product group, the female streamer 1 was selected as the female streamer with significant feminine traits, and the male streamer 1 was selected as a male streamer with significant masculine traits.

In the male product group, the masculinity score of the male streamer 2 was significantly higher than that of the female streamer 2 (*M*_Male streamer 2_ = 6.16, SD_Male streamer 2_ = 0.898; *M*_Female streamer 2_ = 1.92, SD_Female streamer 2_ = 0.997; *p* < 0.01). The femininity scores of the female streamer 2 were significantly higher than those of the male streamer 2 (*M*_Female streamer 2_ = 6.16, SD_Female streamer 2_ = 1.14; *M*_Male streamer 2_ = 1.88, SD_Male streamer 2_ = 1.27, *p*<0.01), while the difference between masculinity and femininity of the other three streamers was not significant. Therefore, in the male product group, the male streamer 2 was selected as the male streamer with obvious masculine traits, and the female streamer 2 was selected as the female streamer with feminine traits.

In addition, there was no significant difference in familiarity, likability, professionalism, trustworthiness and attractiveness of the selected streamers, so we can exclude the possibility that factors other than gender may affect the results of the formal experiment.

### 3.4 Study 1

Study 1 tested the effect of using non-stereotypical gender role endorsement (as opposed to stereotypical gender role endorsement) on consumers’ purchase intentions for female products. For products with female gender images, stereotypical gender role endorsement triggered higher purchase intentions. We then further investigated the moderating role of traditional gender ideology. For consumers with high gender ideology, their purchase intentions are higher for products with stereotypical gender role endorsement compared to non-stereotypical gender role endorsement. For consumers with low traditional gender ideology, there is no significant difference in their willingness to purchase products with the two endorsement modes. In order to exclude the influence of price, brand and other factors, the brand in the two material videos was same, using a real lipstick brand.

#### 3.4.1 Pre-test

The validity of the manipulation of endorsement types (gender stereotypes/counter-gender role stereotypes) was first tested in a pre-test with a total of 46 participants recruited on the Credamo platform (*N* = 46, female 73.9%). Two questions, “For the product in the video, the streamer in the video represents a typical streamer for this product” and “The content of this video reflects a typical female/male scenario” were used to test the subjects’ perceptions of gender stereotypes, examined at a 7-point Likert scale (1 = completely disagree, 7 = completely agree) (Baxter et al., 2016).

The results of the manipulation test showed that Cronbach’s *α* = 0.753 and the reliability was within the acceptable range. In terms of gender stereotype perception, the scores of the gender stereotype group were significantly higher than the counter-gender stereotype group (*M*_gender stereotype_ = 5.36, SD_gender stereotype_ = 0.93; *M*_counter-gender stereotype_ = 4.27, SD_counter-gender stereotype_ = 1.27; *p* < 0.01). This indicated that the experimental manipulation was successful and can be continued to analyze the main effect test for the effect of gender stereotype endorsement types on purchase intentions.

#### 3.4.2 Material and procedure

We recruited participants through the credamo platform. First of all, question “How often do you watch e-commerce live broadcasting” was used as a screening question to test and ensure that all the recruited participants had e-commerce live broadcasting viewing experience. After validity screening, a total of 203 participants were recruited to complete this study (*N* = 203, females 62.6%). Among them, 99 participants were randomly assigned to the stereotypical gender role endorsement group, and the other 104 participants were randomly assigned to the non-stereotypical gender role endorsement group.

All participants first completed the traditional gender ideology measure. Next, the participants were asked to watch an e-commerce live video clip, in which participants of the stereotypical gender role endorsement group watched a video of the female streamer 1 promoting a lipstick product, and participants of the non-stereotypical gender role endorsement group watched a video of the male streamer 1 promoting a lipstick product. Then, participants were asked to complete a purchase intention survey. Finally, information on gender, age, and educational background was collected.

#### 3.4.3 Measurements

In order to measure the participants’ level of gender ideology, participants were required to fill in the traditional gender ideology measurement items (Cheung and Choi, 2016) before watching the video. These measurement items consisted of 6 question items measured on a 7-point Likert scale (1 = completely disagree, 7 = completely agree).

After watching the videos, in order to measure participant’s purchase intentions, they were asked to answer three questions, “How likely are you to buy the product recommended by the streamer,” “Would you be willing to buy the product recommended by this streamer” and “Would you be willing to recommend the product introduced by the streamer to others,” using the 7-point Likert scale (1 = very reluctant, 7 = very willing) (Dodds et al., 1991; Park and Yang, 2010).

#### 3.4.4 Analysis and results

The scores of the 6 items on the traditional gender ideology variable were first summed up to get the interval of [6, 36] for traditional gender ideology, with the median = 14. And then using this median (14) as a dividing line, participants with scores greater than the median were classified as high traditional gender ideology (*M*_high_ = 20.26, SD_high_ = 4.685), while subjects with scores less than or equal to the median were categorized into the low traditional gender ideology group (*M*_low_ = 9.84, SD_low_ = 2.906). Data analysis results showed that there was a significant difference between the high traditional gender ideology group and the low traditional gender ideology group [*t*(164) = −18.968, *p* < 0.01]. Thus, the division of high/low gender ideology group can be used to prepare for the subsequent test of moderating effect.

For the main effects analysis of the female product study, an independent samples *t*-test was used. The results showed that participants in the non-stereotypical gender role endorsement group scored significantly lower on purchase intention than those in the stereotypical gender role endorsement group (*M*_Non-stereotypical gender role endorsement_ = 2.702, SD_Non-stereotypical gender role endorsement_ = 1.031, *M*_Stereotypical gender role endorsement_ = 3.232, SD_Stereotypical gender role endorsement_ = 0.918, *p* < 0.01), the main effect of the endorsement type on the purchase intention has been proven to exist. In other words, for female products, female streamers triggered higher purchase intention in e-commerce live broadcasting compared to non-stereotypical gender role endorsement, in line with the principle of schema consistency. Therefore, hypothesis 1 was supported.

To further test the moderating effect of gender ideology, a univariate analysis of variance (ANOVA) was conducted using SPSS 24.0 statistical software on 2 (type of endorsement: stereotypical gender role endorsement/non-stereotypical gender role endorsement) * 2 (traditional gender ideology level: high/low) conditions, and the results showed that the type of endorsement had a significant effect on purchase intention [*F*(1, 199) = 16.645, *p* < 0.01], which again validated the main effect. After that, the interaction effect between both types of endorsement and traditional gender ideology was significant [*F*(1, 199) = 11.106, *p* < 0.01].

For the high traditional gender ideology group, individuals in the non-stereotypical gender role endorsement group were significantly lower in purchase intention compared to individuals in the stereotypical gender role endorsement group [*M*_Stereotypical gender role endorsement_ = 3.279, SD_Stereotypical gender role endorsement_ = 0.134; *M*_Non-stereotypical gender role endorsement_ = 2.301, SD_Non-stereotypical gender role endorsement_ = 0.132; *F*(1, 199) = 27.078, *p* < 0.01]. The results indicated that Hypotheses 3 and 3a were supported, gender ideology significantly moderated the effect of endorsement type on purchase intention. Individuals with high levels of gender ideology prefered stereotypical gender role endorsement.

**Figure 1:**
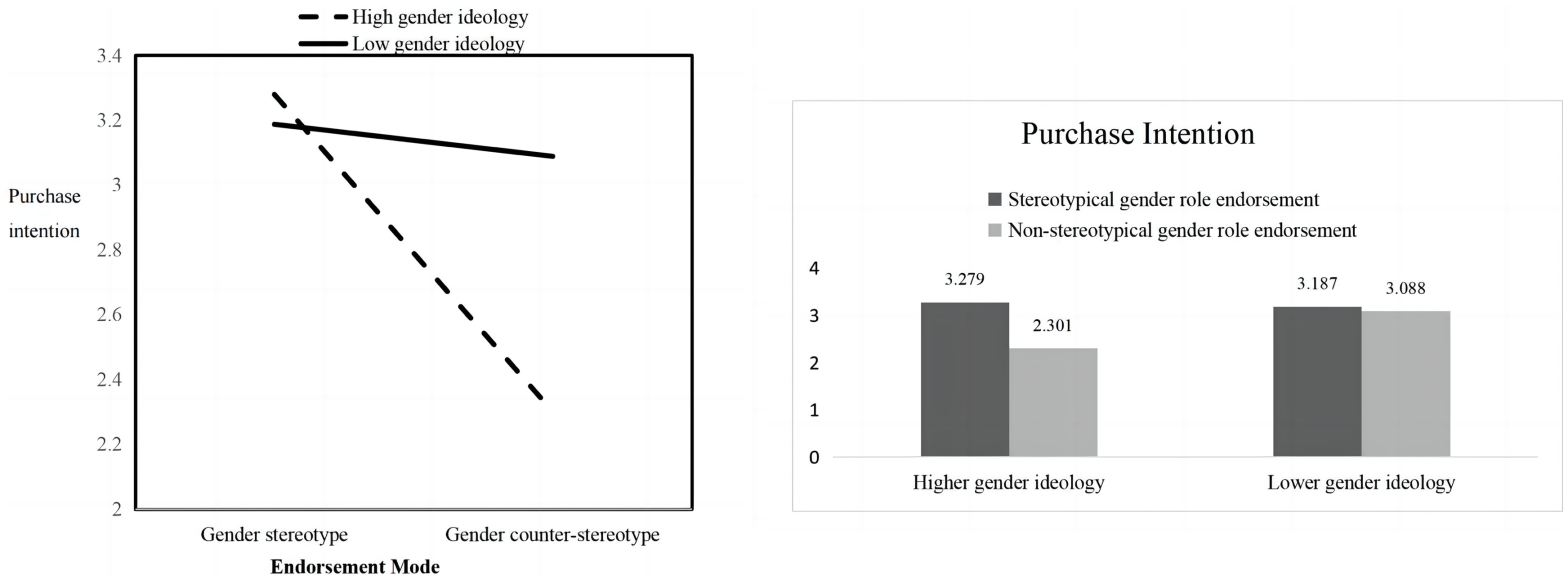
The moderating role of traditional gender ideology in Study 1.

Whereas for the low traditional gender ideology group, there was no significant difference in purchase intention between individuals in the two groups [*M*_Stereotypical gender role endorsement_ = 3.187, SD_Stereotypical gender role endorsement_ = 0.133; *M*_Non- gender role stereotyping endorsement_ = 3.088, SD_Non-stereotypical gender role endorsement_ = 0.129; *F*(1,199) = 0.283, *p* > 0.05]. That is, hypothesis 3b was supported, which was consistent with previous studies. The result is shown in Figure 1.

### 3.5 Study 2

Study 2 repeated the tests of Study 1 with a male product, beer. For male product, the effect of using non-stereotypical gender role endorsement (compared to stereotypical gender role endorsement) on consumers’ purchase intentions was tested. However, there was no significant difference between the effects of the two endorsement modes on consumers’ purchase intentions for male products.

#### 3.5.1 Pre-test

The number of valid participants recruited from the Credamo platform was 48 (*N* = 48, male 39.5%, age from 18 to 50). The measurement item settings of the pre-test were consistent with the pre-test of Study 1. The results of the endorsement type manipulation test showed that the experimental manipulation of the independent variable, endorsement type (stereotypical gender role endorsement/non-stereotypical gender role endorsement), was successful (*M*_Stereotypical gender role endorsement_ = 5.160, SD_Stereotypical gender role endorsement_ = 1.188, *M*_non-stereotypical gender role endorsement_ = 4.000, SD_non-stereotypical gender role endorsement_ = 1.610; *p* < 0.01). Therefore, it is possible to proceed with a main effect analysis of the effectiveness of the non-stereotypical gender role endorsement for male products.

#### 3.5.2 Material and procedure

In the formal experiment, after using the question, “How often do you watch e-commerce live broadcasting,” to screen out participants who had no experience in watching e-commerce live broadcasting, the number of valid participants recruited from the Credamo platform was 130 (*N* = 130, male 37.6%, age from 18 to 60), and all participants had e-commerce live broadcasting viewing experience. Among them, 65 participants were randomly assigned to the stereotypical gender role endorsement group and 65 participants were randomly assigned to the non-stereotypical gender role endorsement group. The process of Study 2 was basically the same as that of Study 1, except that the stereotypical gender role endorsement group watched a video of the male streamer 2 promoting a beer product, and that the non-stereotypical gender role endorsement group watched a video of the female streamer 2 promoting a beer product.

#### 3.5.3 Measurements

Consistent with Study 1 in terms of test screening setting and measurement items for the formal experiment.

#### 3.5.4 Analysis and results

Regarding the main effect test, the independent samples *t*-test analysis showed that there was no significant difference in purchase intention between the stereotypical gender role endorsement group and the non-stereotypical gender role endorsement group (*M*_Stereotypical gender role endorsement_ = 4.497, SD_Stereotypical gender role endorsement_ = 1.392, *M*_Non-stereotypical gender role endorsement_ = 4.667, SD_Non-stereotypical gender role endorsement_ = 1.425, *p* > 0.05). That is, there was no significant difference between the effects of the two types of endorsement on consumers’ purchase intentions for male products. Thus, hypothesis 2 was supported.

## 4 General discussion

### 4.1 Result discussion

Based on schema theory, this study investigated the effect of non-stereotypical gender role endorsement on consumers’ purchase intention in e-commerce live broadcasting, and tested the moderating role of traditional gender ideology in the above process.

Firstly, for female products, stereotypical gender role endorsement triggers higher consumer purchase intentions compared to non-stereotypical gender role endorsement, which is consistent with the conjecture given by Schema Congruity Theory. In the context of live shopping, the congruence between the product and the streamer affects the consumer’s perceived attractiveness and credibility of the streamer and then further increases consumers’ purchase intention (Park and Lin, 2020). When the schema is the gender image of the product, the streamer fits with the gender image of a product in the context of stereotypical gender role endorsement, generating schema congruence (Lynch and Schuler, 1994). That, in turn, can positively influence the product purchase intention. On the contrary, in the non-stereotypical gender role endorsement context, the gender images of a streamer and a product produce incongruent effect. For example, when a male streamer endorses a female product, it may have a negative effect on the purchase intention (Chen et al., 2024). Thus, in order to trigger higher purchase intention, the non-stereotypical gender role endorsement approach should be used cautiously in the e-commerce live broadcasting context of female products.

In addition, in terms of industry reality, according to the Top List of streamers in November 2023 by China Tiktok Feigua Data, for the head streamers who promote beauty and skincare products, female streamers are still dominant at this stage, with men accounting for only 10% of the top 10 streamers. Obviously, male is a very rare label, both for the professional role played by the beauty streamer, or for the endorsement phenomenon in beauty and skin care industry. The reason is that when male head streamers start to promote for beauty and skincare products, it clearly violates male characteristics under traditional gender role model, and thus generated a gender-counter stereotype perception among consumers.

Secondly, for male products, there is no significant difference between the effect of stereotypical gender role endorsement and non-stereotypical gender role endorsement on consumers’ purchase intentions. This is consistent with previous research on traditional marketing channels such as advertising, where there is no significant difference in evaluations of spots in male product advertisements regardless of whether a male or female voice is used (Whipple and McManamon, 2002). This may be due to the fact that for male products, female characteristics such as helpfulness, gentleness, and good listening skills provide a certain amount of warmth (Bye et al., 2022), which will be then transferred to consumers’ perceptions of the product. This neutralizes the negative impact of gender conflict on consumers’ purchase intentions in the non-stereotypical gender role endorsement context to some extent.

Previous studies have shown that the typical features of warm imagery include friendliness, helpfulness, and consideration (Aaker et al., 2010). Female spokespersons are naturally associated with warm imagery (Eagly and Mladinic, 1994), which can enhance their persuasive power (Linne et al., 2022) and then further increase consumers’ purchase intentions (Pogacar et al., 2021).Therefore, even though female endorsement of male products may create a certain degree of cognitive ambivalence among consumers, the warmth perceptions generated by the female streamer may offset the negative effect of cognitive ambivalence to a certain extent.

Thirdly, for consumers with high traditional gender ideology, on the one hand, their purchase intentions for stereotypical gender role endorsement is higher compared to non-stereotypical gender role endorsement. For consumers with low traditional gender ideology, on the other hand, there is no significant difference in purchase intentions for both of the endorsement types. Previous research argued that individuals with more traditional and conservative gender role attitudes will be more receptive and favorable to advertisements that conform to their cognitive patterns than individuals with more advanced and open gender role attitudes. In other words, consumers with high traditional gender ideology will be more attracted to stereotypical advertisements (Morrison and Shaffer, 2003).

The reason is that the higher the traditional gender ideology, the higher the level of gender schematization of a consumer, the more rigid the impression of gender roles, the more inclined to process the advertisement information through the perspective of gender, and therefore has a higher preference for gender role endorsement (Wang and Lianrong, 2005). Empirical studies on live e-commerce have also found that consumers with higher levels of gender ideology pay more attention to messages that are consistent with gender stereotypes (Wenting et al., 2022).

In conclusion, the results of this study indicate that the type of endorsement (non-stereotypical gender role endorsement/stereotypical gender role endorsement) has a significant effect on consumers’ purchase intentions, and that gender ideology plays a moderating role in this effect. It’s worth noting that this effect was only found for products with female gender images and for consumers with high traditional gender ideology.

### 4.2 Result contributions

The theoretical significance of this paper lies in the following: firstly, breaking through traditional marketing channels, we have extended the research on non-stereotypical gender role endorsement and schema theory to the field of e-commerce live broadcasting, which generates certain theoretical significance for expanding the application boundaries of schema theory. Secondly, the introduction of traditional gender ideology as a moderating variable has enriched the existing research on the mechanism of the congruity relationship between spokespersons and products in e-commerce live broadcasting.

Meanwhile, some practical suggestions can be put forward based on this study. On the one hand, for brand owners, when formulating e-commerce live broadcasting marketing plans for female products, they should carefully choose male streamers. On the other hand, for male streamers, the number of female products in their live studios should be increased or decreased based on the live broadcast users’ levels of gender ideology. Therefore, this study has important practical significance for brand owners and streamers to effectively adopt non-stereotypical gender role endorsements to enhance purchase intentions.

In addition, this work extends the discussion of gender and gender stereotypes to celebrity endorsement activities in e-commerce live broadcasting, and the results can have certain social significance. The findings of this study imply that even in the highly innovative scene of e-commerce live studios, consumers’ attitudes toward gender are not as highly inclusive as they are perceived to be. Actions against gender stereotypes still have a long way to go.

### 4.3 Limitations and recommendations for future research

There are certain limitations and room for improvement in this study, mainly in the following three directions:

To begin with, this study takes purchase intention as the dependent variable to study the influence of non-stereotypical gender role endorsement. Nonetheless, with the development of e-commerce live broadcasting, especially the normalization of merchants’ live broadcasting, the role of live broadcasting may gradually develop in the direction of improving the product attitude and brand attitude, etc. Hence, in order to reflect consumers’ evaluation in a more multidimensional way, the subsequent studies can try to take brand awareness, brand favorability and other variables as dependent variables. Besides, more live broadcasting platforms can also be included in future research to provide more beneficial support for the study of non-stereotypical gender role endorsement.

Next, the results of Study 2 show that for male products, the endorsement of female streamer does not lower consumers’ purchase intentions, which can be interpreted as the warmth perceptions embodied in the female image improves consumers’ acceptance of this gender conflict. Nonetheless, the role of related variables such as warmth perception in this study has not been further researched in this study, which needs to be explored and verified by future studies.

Finally, our research design follows the traditional gender dualism, dividing the gender of streamers into male and female, and dividing the gender attributes of products into masculinity and femininity. With the mutual penetration of male and female occupations, the boundaries between male and female gender roles are becoming increasingly blurred. Traditional gender stereotypes are merging, and androgyny has become a trend (Wenting et al., 2022). The proposal of androgyny means that individuals can freely manifest both male and female characteristics without following traditional gender stereotypes (Bem, 1974). Traditional gender dualism hinders the full development of individual personality, while androgyny generates greater adaptability in different situations. Therefore, future research can incorporate androgyny into the gender classification of streamers.

## Data availability statement

The raw data supporting the conclusions of this article will be made available by the authors, without undue reservation.

## Ethics statement

The studies involving humans were approved by South China University of Technology. The studies were conducted in accordance with the local legislation and institutional requirements. The participants provided their written informed consent to participate in this study. Written informed consent was not obtained from the individual(s) for the publication of any potentially identifiable images or data included in this article because all the images used for these experiments are all publicly available on the Taobao e-commerce platform.

## Author contributions

JF: Conceptualization, Funding acquisition, Writing – original draft, Writing – review & editing. SH: Data curation, Writing – original draft. XC: Data curation, Formal analysis, Methodology, Writing – original draft.
